# Down‐regulation of anti‐inflammatory TIPE2 may aggravate adenoidal hypertrophy in children

**DOI:** 10.1002/2211-5463.12821

**Published:** 2020-03-22

**Authors:** Kun Gao, Yanzhong Li, Zhiyong Yue, Jie Han, Xuanchen Zhou, Xiaoting Wang

**Affiliations:** ^1^ Department of Otorhinolaryngology–Head and Neck Surgery Shandong Provincial Hospital affiliated to Shandong University Jinan City China; ^2^ Department of Otorhinolaryngology Qilu Hospital of Shandong University NHC Key Laboratory of Otorhinolaryngology (Shandong University) Jinan City China

**Keywords:** adenoidal hypertrophy, IL‐6, inflammation, tumor necrosis factor‐alpha‐induced protein‐8 like‐2

## Abstract

Adenoidal hypertrophy (AH) is a common disorder in the pediatric population, with common symptoms including mouth breathing, nasal congestion, hyponasal speech, snoring and obstructive sleep apnea. Although the pathogenesis of AH has not been fully elucidated, recent studies have indicated that immune responses may play an important role in AH. Tumor necrosis factor‐alpha (TNF‐α)‐induced protein‐8 like‐2 (TIPE2) is a newly identified protein that negatively regulates the activation of inflammatory pathways. Here, we investigated the effect of TIPE2 in AH in children. We observed that the levels of TNF‐α and interleukin‐6 were greater in the adenoid tissue of AH children than in healthy control subjects (*P* < 0.01), and this increase was positively correlated with the severity of AH. The level of TIPE2 expression was decreased compared with control and was negatively correlated with AH. TIPE2 overexpression in primary human monocytes (isolated from adenoid tissue of children with AH) inhibited the activation of nuclear factor‐κB and the expression of TNF‐α and interleukin‐6. These results suggest that overexpression of TIPE2 may attenuate AH through inactivation of the nuclear factor‐κB signaling pathway.

## Graphical abstract

Here, we report that TIPE2, a protein that maintains immune homeostasis by negatively regulating innate immunity, influences adenoidal hypertrophy of children by inhibiting the activation of nuclear factor‐κB and expression of tumor necrosis factor alpha and interleukin‐6.

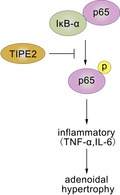

**Abbreviations**
AH: adenoidal hypertrophy
IL‐6: interleukin‐6
NF‐κB: nuclear factor‐κB
OSA: obstructive sleep apnea
TIPE2: tumor necrosis factor‐alpha‐induced protein‐8 like‐2
TNF‐α: tumor necrosis factor alpha

Adenoidal hypertrophy (AH), also known as pharyngeal tonsil hyperplasia, is a common disorder in the pediatric population. Increased nasal obstruction, hindered nasal drainage, and rhinitis and sinusitis secretions stimulate the adenoids to continue to proliferate, forming a vicious circle of mutual causality [1]. The common clinical symptoms include mouth breathing, nasal congestion, hyponasal speech, snoring, obstructive sleep apnea (OSA), chronic sinusitis and otitis media [2]. Clinical practice suggests that the most effective way to treat AH is adenoid surgery. However, due to the special growth stage of children, there is much controversy on adenoid surgery for AH treatment [3]. Therefore, the study of possible targets or therapies is of great value in the treatment of AH.

The pathogenesis of AH has not been fully elucidated, although several triggers (such as smoking, allergic diseases and microbial stimuli) have been confirmed to be associated with the occurrence and development of AH. Recently, many studies have shown that immune responses may play an important role in AH [4]. Adenoids are part of Waldeyer’s ring, a lymphoid structure consisting of the adenoids and palatine tonsils. As the body’s first immune barrier and important effector, Waldeyer’s ring is critical for antigen presentation and local immune response. For example, the level of CD64 in mononuclear cells of adenoid tissues (in patients with AH) is higher than in normal tissues [5]. The proinflammatory mediator [interleukin‐6 (IL‐6)] promotes the expression of vascular endothelial growth factor, which may play a role in promoting AH in children [6].

Tumor necrosis factor‐alpha (TNF‐α)‐induced protein‐8 like‐2 (TIPE2), a recently identified protein, is essential for maintaining immune homeostasis by negatively regulating innate immunity [7]. TIPE2 is mainly expressed in lymphoid tissues, and knockdown of the TIPE2 of mice is more susceptible to multiple organ inflammation. *In vitro* experiments showed that TIPE2 can inhibit the activation of activated protein 1 and nuclear factor‐κB (NF‐κB), and TIPE2 deletion cells overreact to Toll‐like receptor and T‐cell receptor activation [8, 9]. Considering the relationship between TIPE2 and immune response, we hypothesized that TIPE2 may play a role in AH. The aim of the research was to investigate the role of TIPE2 in AH and the detailed mechanism of TIPE2 regulating immune response in patients with AH.

## Materials and methods

### Tissues collection

A total of 48 children with AH (21 females, 27 males; mean age, 6 years) and 7 healthy children collected in Shandong Provincial Hospital affiliated to Shandong University were enrolled in this study. AH was diagnosed by symptoms including nasal congestion, rhinorrhea, snoring, congestion, mouth breathing, sleep apnea, cough, among others. To exclude the influence of different basal inflammations of patients on the results, we simplified the study variables and excluded patients with allergy‐related diseases (such as allergic rhinitis and chronic rhinosinusitis). This study was in accordance with the World Medical Association Declaration of Helsinki [10]. The written informed consent was obtained from patients following the approval of the Ethics Committee of Shandong Provincial Hospital affiliated to Shandong University (Approval Number: LCYJ: NO. 2019‐144). The adenoid tissues were sampled during the adenoidectomy. Each specimen was divided into two parts. One was immediately frozen in liquid nitrogen for western blot or ELISA analysis, and the other was for the isolation of primary monocytes.

### Clinical scores evaluation

The clinical scores were evaluated by the Obstructive Sleep Apnea Questionnaire (OSA‐18), a Likert‐type scoring system that includes five aspects (sleep disturbances, physical symptoms, emotional symptoms, daytime function and caregiver concerns) [11]. The questionnaire consists of 18 items, with each score scale from 1 (none) to 7 (all of the time). The mean value of the 18 items was served as the overall score for each patient.

### Cell culture and transfection

The isolation of primary human monocyte in adenoid tissue was referred from a previous report [4]. In brief, the adenoid tissue homogenate was loaded on a 70‐µm cell strain to get the cell suspension. Adenoidal mononuclear cells were separated by standard density gradient centrifugation (at a density of 1.077 g·mL^−1^). Cells were cultured in RPMI 1640 medium supplemented with 10% FBS and 2 mm glutamine in an incubator (37 °C). Cells were cultured about 80–90% confluent and prepared for transfection. The TIPE2 overexpression was implemented by cell transfection (with the cell density of 10^6^ cells·mL^−1^) with plasmids (PPK5 and PRK5‐TIPE2) by Lipofectamine 2000 according to the manufacturer’s protocols (Invitrogen, Carlsbad, CA, USA). Forty‐eight hours later, transfected cells were collected, and the transfection efficacy was determined by western blot.

### Western blot

Western blot analysis was performed as previously reported [12]. In brief, the adenoid tissues or primary human monocytes were extracted by radioimmunoprecipitation assay lysis buffer supplemented with protease and phosphatase inhibitor. Equal amounts of the protein samples were loaded and separated by SDS/PAGE and transferred to a poly(vinylidene difluoride) membrane. After blocking, the membranes were incubated with primary antibodies [TIPE2 (ab110389; Abcam, Cambridge, MA, USA), NF‐κB p65 (#8242; CST, Danvers, MA, USA), phospho‐NF‐κB p50 (#3033; CST), IκB‐α (#9242; CST), GADPH (#5174; CST)] at dilution 1 : 1000. Then the membranes were incubated with horseradish peroxidase‐conjugated secondary antibodies and visualized with a chemiluminescence imaging system according to the manufacturer’s instructions. The density of the bands was normalized to each GADPH.

### ELISA

The levels of proinflammatory mediators of adenoid tissue homogenate or total protein extracts of primary human monocytes were measured by a commercially available ELISA kit (R&D Systems, Minneapolis, MN, USA) according to the manufacturer’s suggestion.

### Statistical analysis

For statistical analysis, we used Student’s *t*‐test of samples by spss software (New York, NY, USA). The *P* values <0.05 were considered a significant difference.

## Results

### The levels of TNF‐α and IL‐6 are positively correlated with AH

**Fig. 1:**
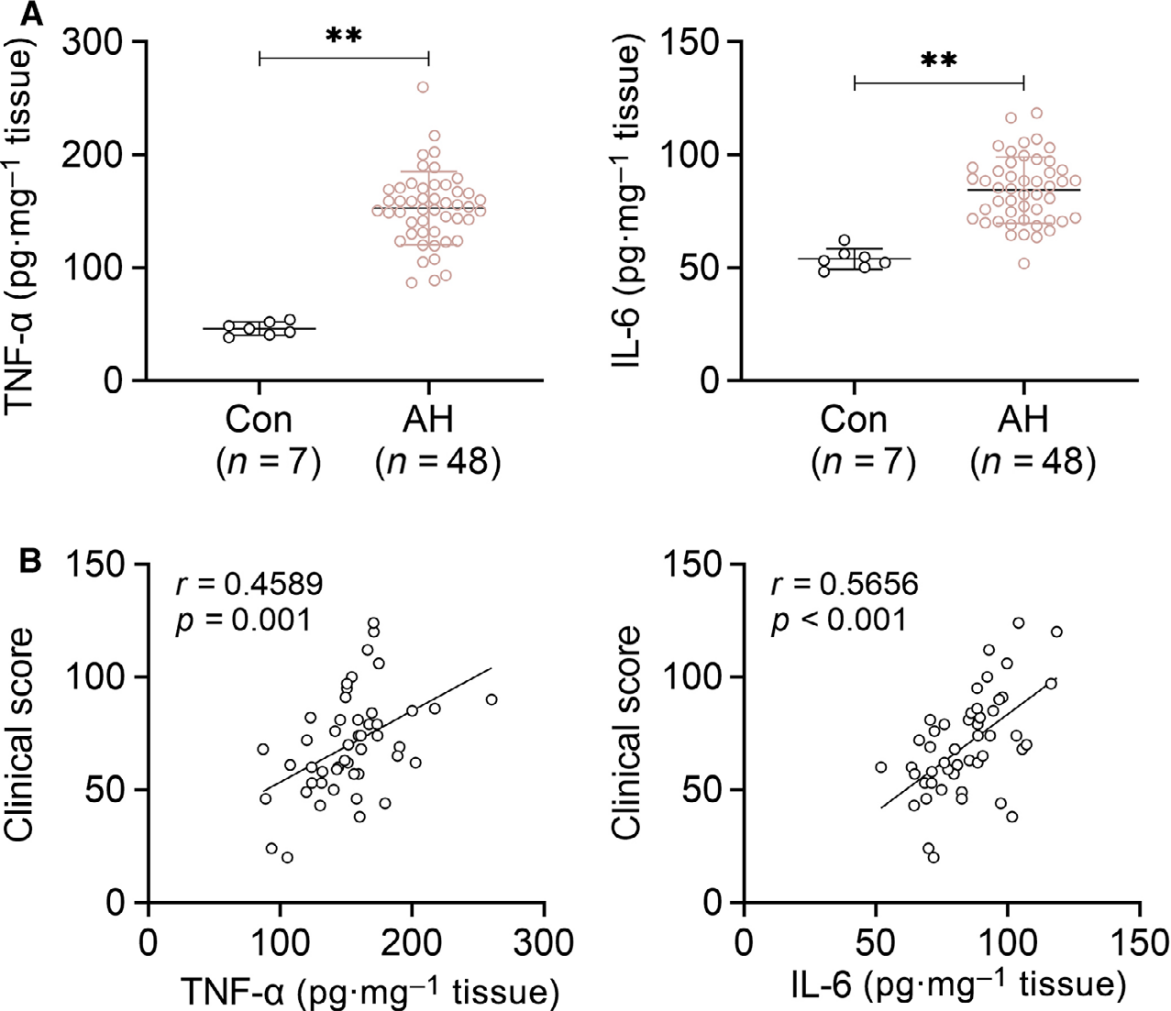
The levels of TNF‐α and IL‐6 are positively correlated with AH. (A) The levels of TNF‐α and IL‐6 in adenoid tissues of children with AH (*n* = 48) or healthy children (*n* = 7) determined by ELISA. (B) Correlation between TNF‐α or IL‐6 concentration and the patients’ clinical scores. Student’s *t*‐tests were used as statistical analysis by spss software.

Fifty‐five children were enrolled in this research: 48 patients with AH and 7 healthy children. The levels of TNF‐α and IL‐6 in adenoid tissue of healthy children or children with AH are shown in Fig. 1A. The results showed that the levels of TNF‐α and IL‐6 in adenoid tissue of patients were higher compared with controls (*P* < 0.01). The correlation of TNF‐α and IL‐6 with clinical score is shown in Fig. 1B. The regression analysis showed that the levels of TNF‐α and IL‐6 are positively correlated with AH clinical score (TNF‐α: *r* = 0.4589; IL‐6: *r* = 0.5656). The results indicated that the higher the clinical severity of AH, the higher the levels of TNF‐α and IL‐6.

### The level of TIPE2 is negatively correlated with AH

**Fig. 2:**
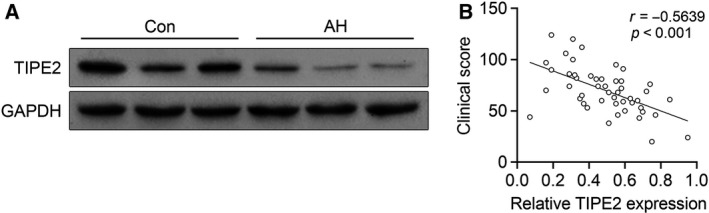
The level of TIPE2 is negatively correlated with AH. (A) Levels of TIPE2 in adenoid tissues of children with AH (*n* = 48) or healthy children (*n* = 7) determined by western blot. (B) Correlation between TIPE2 expression and the patients’ clinical scores.

We observed that the level of TIPE2 in adenoid tissue of patients was significantly decreased compared with nontumor tissues (Fig. 2A). Besides, the correlation of TIPE2 with clinical score is shown in Fig. 2B. The regression analysis showed that the level of TIPE2 is negatively correlated with AH clinical score (*r* = 0.5639).

### TIPE2 regulates the activation of NF‐κB and expression of TNF‐α and IL‐6 in monocytes in adenoid tissue

**Fig. 3:**
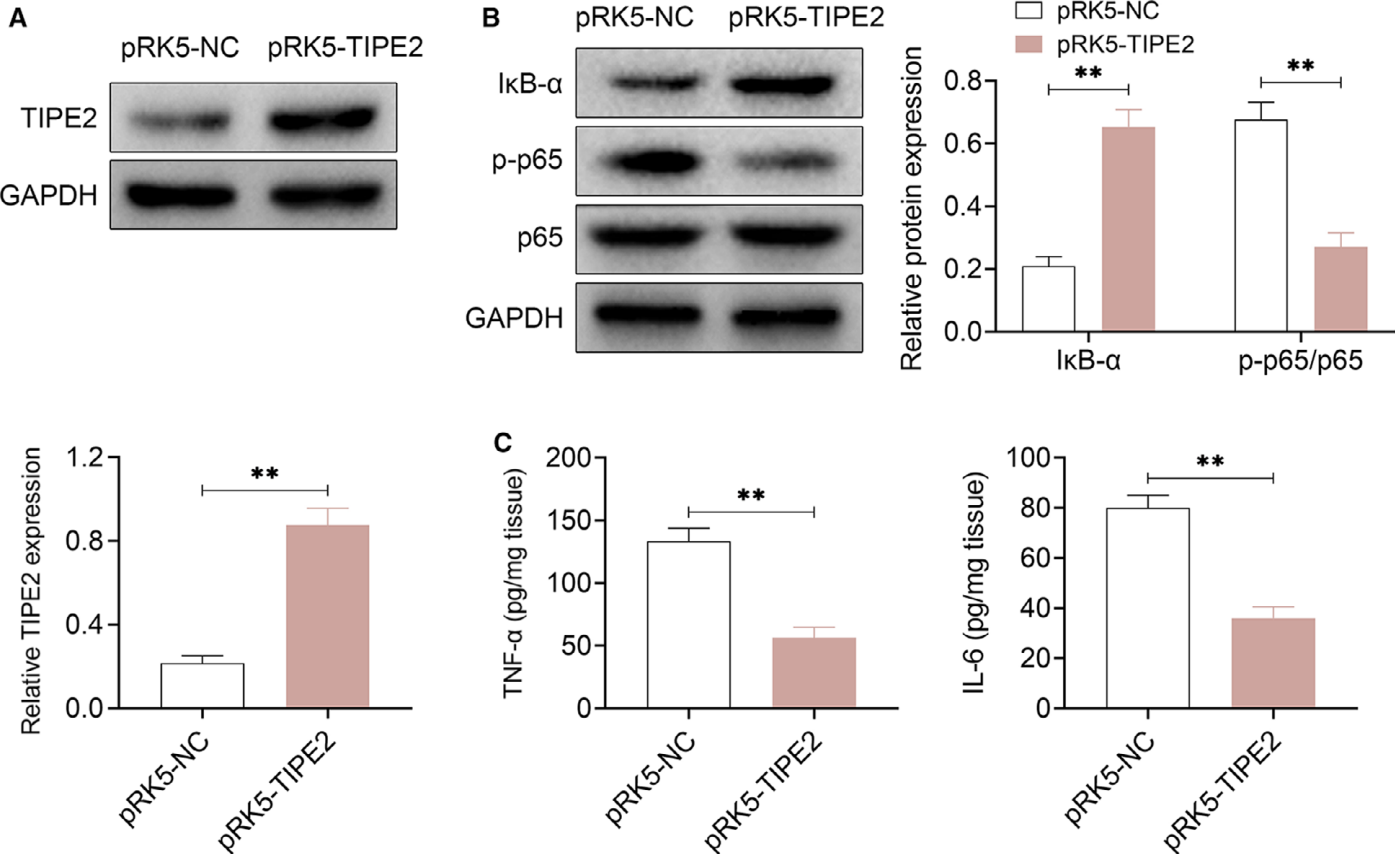
TIPE2 regulates the activation of NF‐κB and expression of TNF‐α and IL‐6 in monocytes in adenoid tissue. (A) The transfection efficacy of TIPE2 plasmids (pRK5‐TIPE2) or negative control (pRK5‐NC) analyzed by western blot. (B) The expression of p‐p65, p65 and IκB‐α in human primary monocytes analyzed by western blot. (C) The levels of TNF‐α and IL‐6 in human primary monocytes analyzed by ELISA (*n* = 3). The data were shown as mean ± standard deviation. Student’s *t*‐tests were used as statistical analysis by spss software. **P < 0.05 versus pRK5‐NC group.

To investigate the effect of TIPE2 in the activation of the inflammatory signaling pathway (NF‐κB) and monocytes, the TIPE2‐overexpressed monocytes (isolated from adenoid tissue of patients with AH with low TIPE2 expression) were established. As shown in Fig. 3A, the expression of TIPE2 in pRK5‐TIPE2 cells was increased significantly compared with the negative control (*P* < 0.01), which indicated that the TIPE2 overexpression cells were successfully established. Furthermore, we detected the expression of some key proteins of the NF‐κB signaling pathway (Fig. 3B). The ratio of phosphorylated p65 to total p65 (p‐p65/p65) was decreased in TIPE2 overexpression cells compared with control, whereas IκB‐α was increased in TIPE2 overexpression cells (*P* < 0.01). The results indicated that TIPE2 overexpression induced the inhibition of the NF‐κB signaling pathway. Furthermore, the levels of TNF‐α and IL‐6 presented lower in TIPE2 overexpression cells compared with negative control (*P* < 0.01). These data suggest that the TIPE2 overexpression could inhibit the activation of the NF‐κB signaling pathway, as well as the expression of the proinflammatory mediators.

## Discussion

As the body’s first immune barrier and important effectors, adenoids are critical for local immune response to stimulus or pathogens [13]. The relationship of inflammation and AH is not fully clear. However, many studies have demonstrated the effects of different kinds of immunocytes in AH or allergic inflammation in adenoidal tissue. Several studies revealed that numerous IgE mast cells were found in adenoidal tissue of allergic children [14, 15]. Besides, the level of IgA receptors on eosinophils is increased in patients with allergies [16]. Moreover, recently, many reports have demonstrated that other populations of lymphoid cells were related with AH by regulating the immune response, such as B lymphoid regulatory cells and follicular T helper lymphoid cells [17]. Some serum inflammatory mediators also served as the markers of AH in children. For example, overexpression of CD163, a typical marker of monocyte/macrophage activation, could indicate the severity of AH [18]. A high level of serum myeloperoxidase, a typical marker of neutrophils activation, was observed in recurrent lower respiratory infection of children [19]. Our present research showed that the levels of tissue TNF‐α and IL‐6, two main proinflammatory mediators, were increased markedly in children with AH and were positively related to the clinical scores. Our results and the previous research all indicated that the coexisting allergic disorder or a chronic, severe and recurrent inflammatory process is associated with AH.

NF‐κB, a nuclear transcription factor, regulates a large number of genes that are critical for the regulation of inflammation, apoptosis, tumorigenesis and various autoimmune diseases. NF‐κB is activated by a variety of stimuli, including growth factors, cytokines, pharmacological agents and stress [20]. TIPE2 was a recently identified protein that can inhibit the activation of activated protein 1 and NF‐κB, and thus is essential for maintaining immune homeostasis by negative regulation of innate immunity [7, 8, 9]. So far, few reports have demonstrated the relationship between TIPE2 and AH. Considering the relationship between TIPE2 and the immune response, we investigated its expression in patients with AH and the possible mechanism. This research showed that the level of TIPE2 in adenoid tissue of patients was significantly decreased compared with the healthy children. Besides, the level of TIPE2 is negatively correlated with AH severity. In addition, *in vitro* study demonstrated that TIPE2 overexpression in monocytes/macrophages of patients with AH inhibited the activation of the NF‐κB signaling pathway, as well as the expression of proinflammatory mediators (TNF‐α and IL‐6). Our research confirmed that TIPE2 is related to AH of children and could be a new therapeutic target for treatment of AH and its related diseases, although more research is needed to verify the role of TIPE2 in immune response of AH and more detailed mechanisms. For example, the *in vivo* effects of TIPE2 knockdown or overexpression need to be confirmed. Although the negative regulatory effect of TIPE2 on NF‐κB was known, the specific regulatory processes and effects of TIPE2 on NF‐κB need further experiments.

## Conclusion

Our research first reported that TIPE2 is related with AH of children and that TIPE2 could be a new therapeutic target for the treatment of AH and its related diseases.

## Conflict of interest

The authors declare no conflict of interest.

## Author contributions

YL and ZY conceived and designed the project. KG and JH analyzed and interpreted the data. XZ and XW wrote the paper.

## Data Availability

All data generated or analyzed during this study are included in this published article.
